# Triglyceride–Glucose Index as a Potential Indicator of Sarcopenic Obesity in Older People

**DOI:** 10.3390/nu15030555

**Published:** 2023-01-20

**Authors:** Bokun Kim, Gwonmin Kim, Yongkook Lee, Keisuke Taniguchi, Tomonori Isobe, Sechang Oh

**Affiliations:** 1 Department of Anti-Ageing Health Care, Changwon National University, Changwon 51140, Republic of Korea; 2 Future Convergence Research Institute, Changwon National University, Changwon 51140, Republic of Korea; 3 Medical Research Institute, Pusan National University, Busan 46241, Republic of Korea; 4 Department of Leisure Sports, Seoil University, Seoul 02192, Republic of Korea; 5 Department of Physical Therapy, AHRU Medical Care and Welfare Professional Training College, Tsuchiura 300-0032, Japan; 6 Faculty of Medicine, University of Tsukuba, Tsukuba 305-8575, Japan; 7 Faculty of Rehabilitation, R Professional University of Rehabilitation, Tsuchiura 300-0032, Japan

**Keywords:** insulin resistance, obesity, sarcopenia, sarcopenic obesity, older population, TyG index

## Abstract

Purpose: This population-based cross-sectional study aimed to determine whether the triglyceride–glucose index (TyG index) is associated with sarcopenic obesity (SO) and whether it would be a helpful indicator of SO. Methods: A total of 3821 participants aged ≥ 60 years were selected for the study group, and 4919 participants aged 20–39 years were included as a reference group. The participants were allocated to sarcopenia, obesity, and SO groups depending on if their body mass index (BMI) was ≥25 kg/m^2^ and their sarcopenia index was ≤1 standard deviation (SD) lower than the mean of the reference group. The sex-specific differences and trends among the participants were analyzed by using the TyG index tertiles, and appropriate cut-off values of the TyG index for SO were calculated. Results: As the TyG index increased, BMI increased, but the sarcopenia index decreased in both sexes. Males and females in the middle and highest tertiles of the TyG index were 1.775 and 3.369, and they were 1.993 and 3.157 times more likely to have SO, respectively. The cut-off values of the TyG index for SO in males and females were ≥8.72 and 8.67, respectively. Conclusion: A high TyG index is positively associated with SO, and the TyG index may be considered a potential indicator of SO.

## 1. Introduction

Sarcopenic obesity is characterized by both high adiposity and low muscle mass [1]. It is associated with a higher risk of several metabolic disorders, including diabetes and cardiovascular diseases [2,3,4]. Compared to those associated with either sarcopenia or obesity alone, the consequences of sarcopenic obesity are more severe and are associated with significantly higher healthcare costs [5,6]. Moreover, because sarcopenic obesity progresses slowly, the early stages of the condition do not receive sufficient attention, which leads to a delay in diagnosis and significant consequences with respect to quality of life and all-cause mortality [3,6,7]. Therefore, older adults should undergo screening to facilitate the early detection and care of patients with sarcopenic obesity to prevent its progression.

Previous studies have shown that both aging and obesity are closely related to the development of insulin resistance [4]. Insulin resistance is associated with high plasma glucose and triglyceride concentrations because of impairments in cellular glucose uptake and utilization and greater hepatic triglyceride secretion [8,9,10,11,12,13] This results in further fat accumulation and an acceleration of muscle catabolism. Thus, there is evidence that insulin resistance and sarcopenic obesity are closely related.

The homeostasis model assessment of insulin resistance (HOMA-IR) has been used for many years to evaluate insulin sensitivity in clinical practice [14]. The triglyceride–glucose index (TyG index) has more recently been introduced and has been shown to significantly correlate with hyperinsulinemic–euglycemic clamp data obtained in Korea, Mexico, and Brazil [15,16,17]. In addition, the TyG index has been shown to be superior to the HOMA-IR for the identification of several insulin-resistance-related conditions, including type 2 diabetes mellitus, arterial stiffness, and non-alcoholic fatty liver disease [18,19,20]. Thus, the TyG index represents a reliable and valid indicator of insulin resistance.

Depending on those considerations, we performed a population-based, world-first cross-sectional study to determine whether the TyG index is related to sarcopenic obesity and whether it would be suitable for use as an indicator of sarcopenic obesity.

## 2. Materials and Methods

### 2.1. Study Design and Participants

**Figure 1:**
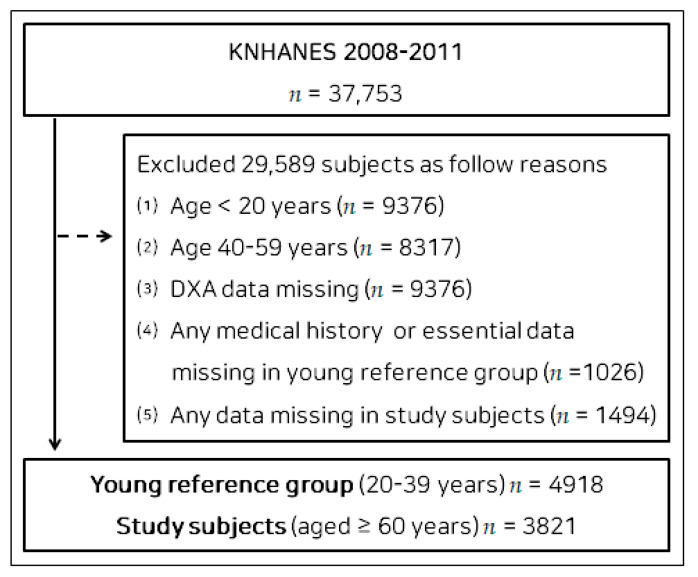
Flow diagram of participant enrollment. Abbreviations: KNHANES, Korea National Health and Nutritional Examination Survey; DXA, Dual X-ray Absorptiometry.

We used a database containing data regarding the general health, nutritional status, and lifestyle of Korean people collected during the Korea National Health and Nutritional Examination Survey (KNHANES) of 2008–2011 for the present study. KNHANES, which was initiated in 1984, is an ongoing national surveillance system assessing Koreans’ health and nutritional status. Of the 37,753 participants in the KNHANES of 2008–2011, 29,589 participants were excluded for the following reasons: (1) age of <20 years (*n* = 9376); (2) age of 40–59 years (*n* = 8317); (3) missing Dual X-ray Absorptiometry (DXA) data (*n* = 9376); (4) any missing medical history or essential data in the young reference group (*n* = 1026); (5) any missing data for study subjects (*n* = 1494). Finally, 4918 participants aged 20–39 years were selected to form a young reference group (Supplementary Materials Table S1), and 3821 participants (1636 males and 2185 females) aged ≥ 60 years were selected as the study group. A flow diagram of participant recruitment is shown in Figure 1. All of the participants provided their written informed consent. The study was performed in accordance with the principles of the Declaration of Helsinki and approved by the Institutional Review Board of Changwon National University (7001066-202207-HR-051).

### 2.2. Sarcopenic Obesity

The heights of the participants were measured without shoes in a standing posture to the nearest 0.1 cm, and their body masses were measured to the nearest 0.1 kg by using digital electronic scales while they were in light clothing. The body mass index (BMI) was calculated as body mass (kg)/height (m)^2^. Obesity was defined as a BMI of ≥25 kg/m^2^ according to the published criterion for inhabitants of the Asia–Pacific region [21].

DXA densitometry (Discover-W fan-beam densitometer; Hologic, Marlborough, MA, USA) was used to assess the participants’ body composition. The Hologic software was utilized to analyze the assessments by computing estimations of lean, fat, and bone mass (in kilograms). Extended analysis was conducted to sort the body composition results into arms, legs, and trunk by computing data on the fat, lean, and bone mass for each body part. For the sarcopenia index, appendicular skeletal muscle mass (ASM) was calculated as the arm and leg lean mass minus the arm and leg bone mass [22,23,24], then divided by the body mass, and it was expressed as a percentage ((ASM/body mass) × 100). Sarcopenia was defined as a sarcopenia index at least one standard deviation (SD) below the sex-specific mean of the young reference group (*n* = 4918) according to the method of Janssen et al. [6,23,25]. The calculated cut-off values were 30.18% and 23.75% for males and females, respectively. Participants in the young reference group with a history of serious diseases, such as cancer, stroke, other cardiovascular diseases, or arthritis, were excluded to avoid any influences on the reference values and reduce the amount of missing data.

According to the stated criteria for the diagnosis of obesity and sarcopenia, the participants were allocated to one of three groups: sarcopenia but no obesity (sarcopenia group), no sarcopenia but obesity (obesity group), and the sarcopenic obesity group.

### 2.3. TyG Index

Blood samples were obtained from the antecubital vein from each subject in the morning after a fast of >8 h. The circulating concentrations of glucose, total cholesterol, high-density lipoprotein cholesterol (HDLC), triglyceride, and creatinine, as well as the circulating activities of aspartate aminotransferase and alanine aminotransferase, were measured with enzymatic methods by using a Hitachi automatic analyzer 7600 (Tokyo, Japan). The TyG index was calculated as ln [triglyceride (mg/dL) × fasting plasma glucose (mg/dL)]/2 [16].

### 2.4. Other Parameters and Covariates

The blood pressures of the participants were manually measured three times in a mobile health check-up vehicle, and the mean values were recorded. Parameters that are known or suspected to be associated with sarcopenic obesity were included as potential confounding factors: nutrition, medication, medical history, moderate-to-vigorous physical activity (MVPA), household income, educational level, alcohol consumption, and smoking [26]. Nutritional data were collected by utilizing a food frequency questionnaire composed of 63 food items that are critical sources of energy and nutrients. The questionnaire was designed as an open-ended survey for reporting a variety of dishes and foods by utilizing the 24 h recall method with various measuring aids [27]. The use of medication by and the medical history of the participants were recorded on the basis of a self-reported diagnosis by a physician or prescription, respectively. MVPA was evaluated by using the short form of the International Physical Activity Questionnaire. The reliability and validity of the questionnaire were verified in 12 nations and have been globally employed to evaluate physical activity in recent years [28]. Household income was classified by using tertiles. Self-reported alcohol consumption was categorized as never, ≤1 time/week, 2–3 times/week, or ≥4 times/week; educational level was classified as primary, middle and high school, or college and beyond; smoking habits were classified as never, former, or current smoker. Overall and sex-specific characteristics and comparisons of the study subjects are presented in Supplementary Materials Table S2, and the sex-specific differences and trends of participants by TyG index tertile are listed in Supplementary Materials Table S3. 

### 2.5. Statistical Analysis

Statistical analyses were performed by using the SPSS software, version 20.0 (IBM Corp., Armonk, NY, USA). The data are presented as the mean ± standard deviation or odds ratio and 95% confidence interval (CI). An independent t-test or Mann–Whitney U test was used to compare the datasets between males and females. One-way ANOVA was used to compare the mean values for anthropometric parameters among the three groups, and the Bonferroni post-hoc test was used when the ANOVA showed a significant difference. The Mann–Whitney U test was used for non-normally distributed data that were verified by utilizing Levene’s test for equality of variances. The Jonckheere–Terpstra test was used to identify trends in parameters among the three groups (two-tailed) [29,30,31,32,33]. This test generated standardized statistics that represented the strength of trends in parameters that increased or decreased across groups [29,30,31,32,33]. Logistic regression was employed to evaluate the relationships of the TyG index, which was an independent parameter, with obesity, sarcopenia, and sarcopenic obesity, which were dependent parameters. The fully adjusted model was adjusted for the potential confounders of age, educational level, household income, medication, smoking, alcohol consumption, moderate-to-vigorous physical activity, nutrition, and medical history. The optimal cut-off values of the TyG index for male and female participants for the identification of sarcopenic obesity were derived from receiver operating characteristic (ROC) curves; the area under the ROC curve (AUC), sensitivity, and specificity were calculated. This analysis was conducted by using MedCalc for Windows ver. 9.1.0.1 (MedCalc^®^ Corp, Mariakerke Ostend, Belgium). *p* < 0.05 was accepted as indicating statistical significance.

## 3. Results

Table 1 lists the characteristics of the study participants and the differences between males and females. The mean TyG indexes was 8.70, 8.71, and 8.70 (SD: 0.53, 0.53, and 0.52) for the entire group and for the males and females. The mean sarcopenia index and BMI were 27.83, 27.88, and 27.79 (4.28, 4.32, and 4.25) and 23.8 (3.2, 3.1, and 3.2), respectively. The mean age was 69.2 (6.0), 69.1 (5.9), and 69.4 (6.1), respectively; the mean height was 158.0 (8.9), 158.3 (8.8), and 157.7 (9.0), respectively; the mean body mass was 59.6 (10.0), 59.8 (9.7), and 59.5 (10.3); the mean ASM was 16.61 (3.92), 16.70 (3.88), and 16.55 (3.95), respectively; the mean glucose amount was 101.8 (21.5), 102.2 (22.2), and 101.5 (21.0), respectively; the mean triglyceride amount was 135.4 (68.9), 135.4 (69.0), and 135.4 (68.9), respectively. There were no differences between the sexes concerning these eight parameters, except for height. Supplementary Materials Table S2 lists additional characteristics of the participants. 

The sex-specific differences and trends in the characteristics of the participants according to the tertile of the TyG index are presented in Table 2. In males, the trend test revealed a significant increase in BMI from the lowest to highest tertile of the TyG index (standardized statistic (SS): 8.43; *p* < 0.001); the opposite trend was identified concerning the sarcopenia index (−6.30; *p* < 0.001). Post-hoc testing demonstrated significant differences among the three groups concerning BMI, which increased from the lowest to the middle and highest tertiles of the TyG index. The sarcopenia indexes of the middle and highest tertiles did not differ, but they were significantly lower than that of the lowest tertile. For body mass, fasting plasma glucose (FPG), and triglycerides (TG), the same results as those of BMI in post-hoc and trend testing were found, but no significant results were found for age, height, and ASM. 

In females, the trend test demonstrated a significant trend of BMI increasing from the lowest to the highest tertile of the TyG index (12.73; *p* < 0.001). Post-hoc testing demonstrated significant differences among the three groups concerning BMI, which increased from the lowest to the middle and highest tertiles of the TyG index. However, the opposite trend was identified for the sarcopenia index (−6.46; *p* < 0.001). Post-hoc testing revealed the same results as those for males. For body mass, FPG, and TG, the same results as those of males in post-hoc and trend testing were found, but no significant results were found for age and height. The ASM values of the lowest and middle tertiles did not differ, but they were significantly lower than that of the highest tertile. This parameter showed a significant increasing trend (3.56; *p* < 0.001). Supplementary Materials Table S3 shows the analyses of additional parameters in male and female participants.

**Figure 2:**
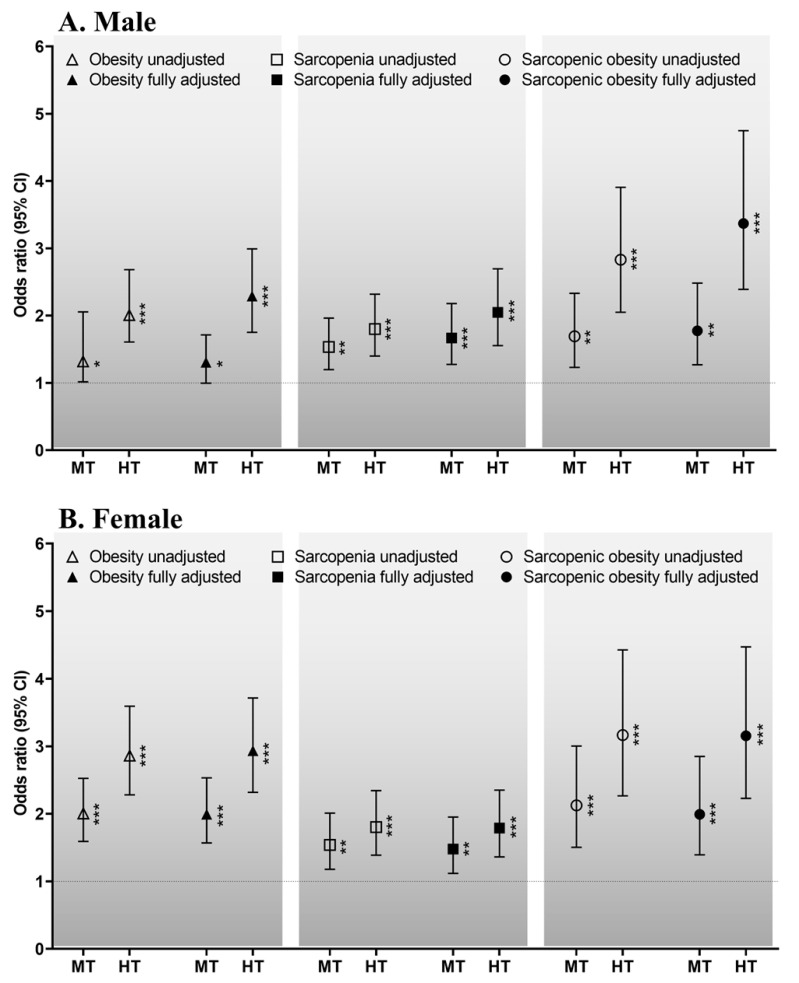
Sex-specific ORs for the relationship of the TyG index with obesity, sarcopenia, and sarcopenic obesity for males (**A**) and females (**B**). Dotted line: reference; solid line: 95% confidence interval (CI); white or black triangles, rectangles, and circles: ORs. * *p* < 0.05, ** *p* < 0.01, *** *p* < 0.001 for the ORs for obesity, sarcopenia and sarcopenic obesity, compared with the lowest tertile. The potential confounders: age, educational level, household income, medication, smoking, alcohol consumption, moderate-to-vigorous physical activity, nutrition, and medical history. Abbreviations: HT, highest tertile; MT, middle tertile; OR, odds ratio; TyG index, triglyceride–glucose index.

The sex-specific odds ratios for the relationships of the TyG index with obesity, sarcopenia, and sarcopenic obesity are shown in Figure 2. The sex-specific odds ratios for the relationships between the TyG index and obesity in males (A) are as follows. In the unadjusted and fully adjusted models, compared with the lowest tertile, the highest and middle tertiles of the TyG index had odds ratios (95% confidence intervals (CIs)) of 2.077 (1.609–2.682, *p <* 0.001) and 1.319 (1.016–2.055, *p <* 0.05) and of 2.290 (1.752–2.992, *p <* 0.001) and 1.306 (0.996–1.714, *p <* 0.05), respectively. For females (B), in the unadjusted and fully adjusted models, compared with the lowest tertile, the highest and middle tertiles had odds ratios of 2.863 (2.281–3.594, *p <* 0.001) and 2.005 (1.591–2.526, *p <* 0.001) and of 2.936 (2.319–3.716, *p <* 0.001) and 1.994 (1.570–2.533, *p <* 0.001), respectively.

The sex-specific odds ratios for the relationships between the TyG index and sarcopenia for males (A) were as follows. In the unadjusted and fully adjusted models, compared with the lowest tertile, the highest and middle tertiles had odds ratios of 1.801 (1.400–2.318, *p <* 0.001) and 1.534 (1.198–1.964, *p <* 0.01) and of 2.048 (1.555–2.696, *p <* 0.001) and 1.668 (1.276–2.180, *p <* 0.001), respectively. For females (B), in the unadjusted or fully adjusted models, compared with the lowest tertile, the highest and middle tertiles had odds ratios of 1.804 (1.388–2.344, *p <* 0.001) and 1.540 (1.179–2.012, *p <* 0.01) and of 1.790 (1.362–2.352, *p <* 0.001) and 1.478 (1.118–1.953, *p <* 0.01), respectively.

The sex-specific odds ratios for the relationships between the TyG index and sarcopenic obesity for males (A) were as follows. In the unadjusted and fully adjusted models, compared with the lowest tertile, the highest and middle tertiles had odds ratios of 2.830 (2.050–3.907, *p <* 0.001) and 1.694 (1.231–2.331, *p <* 0.01) and of 3.369 (2.390–4.748, *p <* 0.001) and 1.775 (1.269–2.483, *p <* 0.01), respectively. For females (B), in the unadjusted and fully adjusted models, compared with the lowest tertile, the highest and middle tertiles had odds ratios of 3.167 (2.266–4.425, *p <* 0.001) and 2.127 (1.505–3.005, *p <* 0.001) and of 3.157 (2.230–4.469, *p <* 0.001) and 1.993 (1.393–2.851, *p <* 0.001), respectively.

**Figure 3:**
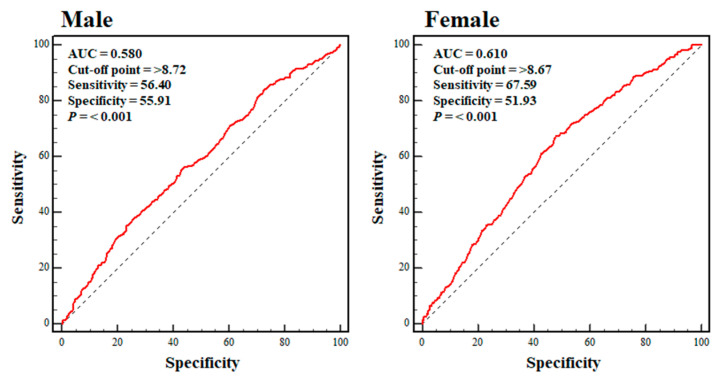
Sex-specific ROC curves pertaining to the TyG index for the sarcopenic obesity group. Dotted blue line: reference; solid red line: AUC, indicative of the accuracy of the use of the TyG index for the identification of sarcopenic obesity; cut-off value: the value of the TyG index that predicts sarcopenic obesity; sensitivity: the probability of individuals who actually have sarcopenic obesity being identified as having sarcopenic obesity; specificity: the probability of individuals who do not have sarcopenic obesity being identified as not having sarcopenic obesity. Abbreviations: AUC, the area under the curve; ROC, receiver operating characteristic.

Figure 3 shows the ROC curves for the TyG index in the male and female participants in the sarcopenic obesity group. The highest sensitivity and specificity values were achieved at a TyG index of ≥8.72 (sensitivity: 56.40%; specificity: 55.91%) and 8.67 (sensitivity: 67.59%: specificity 51.93%) for the male and female participants, respectively (*p* < 0.001 for both). The sensitivity and specificity values in the male participants were close to the low threshold, and those in the female participants were at the low threshold. 

## 4. Discussion

Excessive energy intake, physical inactivity, insulin resistance, oxidative stress, chronic inflammation, and chronic disease are all risk factors for sarcopenic obesity [26]. Of these, insulin resistance is thought to be the most significant Bastard et al. and Dyck et al. showed that proinflammatory molecules induce obesity-associated insulin resistance via crosstalk between cytokine receptors and insulin receptor signaling pathways in animal and human studies [34,35]. In addition, Goodpaster et al. showed that intramuscular fat infiltration induces insulin resistance in individuals with obesity [36,37]. Moreover, DeFronzo and Tripathy and Srikanthan and Karlamangla showed that because skeletal muscle is the largest insulin-sensitive tissue, comprising almost 40% of body mass, its loss is also associated with insulin resistance [13,38]. Thus, obesity-induced insulin resistance that develops during aging promotes the aging-related loss of muscle mass and vice versa; insulin resistance may, therefore, be associated with the deleterious changes in body composition that occur during aging.

In the present study, our first result of an increase in obesity and a decrease in the index of sarcopenia in both sexes as the TyG index increased was observed. In addition, the male and female participants in the middle and highest tertiles of the TyG index were 1.775 and 3.369 or 1.993 and 3.157 times more likely to have sarcopenic obesity, respectively. These odds ratios are higher than those for obesity or sarcopenia alone (Figure 2), suggesting that, together, obesity and sarcopenia are a more potent inducer of insulin resistance than either obesity or sarcopenia alone. Thus, not only obesity-associated insulin resistance, but also sarcopenia-induced insulin resistance is associated with a variety of chronic diseases, such as non-alcoholic fatty liver disease, kidney disease, and type 2 diabetes mellitus; sarcopenic obesity can further increase the prevalence of these diseases through a further exacerbation of insulin resistance [19,30,39,40,41]. Therefore, it is important that older adults are screened to facilitate early detection and care aimed at preventing the progression of sarcopenic obesity.

The analysis of the ROC curves showed that the optimal cut-off values for the TyG index for the prediction of sarcopenic obesity were ≥8.72 and 8.67 in males and females, respectively. No previous study had investigated the relationship between the TyG index and sarcopenic obesity or identified appropriate cut-off values for the TyG index for the identification of sarcopenic obesity. Therefore, further studies are required to verify the validity and reliability of the calculated values in other populations. However, in 2016, Lee et al. reported that middle-aged Korean people with a TyG index ≥ 8.8 were at a higher risk of diabetes than those with values below this cut-off, which suggests that it might be associated with the development of insulin resistance [42]. In 2017, Moon et al. reported that a TyG index ≥ 8.35–8.55 was associated with metabolic syndrome in Korean teenagers [43], which implied that values above this range were also associated with insulin resistance. Considering the influences of age and sex, it is not possible to directly compare the cut-off values calculated in previous studies and the present study. However, it is thought that a TyG index ≥ 8.4–8.5 may indicate the presence of insulin resistance, regardless of age and sex. Thus, the values of the TyG index of ≥8.72 and 8.67 may be considered a potential indicator of sarcopenic obesity.

There were both strengths and limitations to the present study. Significant potential covariates—including demographic and lifestyle factors—that may have affected the relationship between the TyG index and sarcopenic obesity were controlled for in the present study. However, it is unclear whether the findings are applicable to patients of other ethnicities or in other countries because all of the participants were older Korean individuals. In particular, the AUCs (sensitivity and specificity) yielded by the ROC curve analysis were only 0.580 (sensitivity: 56.40%; specificity: 55.91%) and 0.610 (sensitivity: 67.59%; specificity: 51.93%) for males and females, respectively, which casts some doubt on the validity and reliability of the calculated cut-off values. Thus, further studies of patients of other ethnicities should be performed to determine the model’s reproducibility. The results related to the present study’s ROC curve analysis may be utilized as a reference for future studies. 

## 5. Conclusions

This cross-sectional study confirmed that a high TyG index is associated with a higher risk of sarcopenic obesity, and the TyG index may be considered a potential indicator of sarcopenic obesity on the basis of the following findings. First, there was a significant increase in obesity and a significant decrease in the index of sarcopenia in both sexes as the TyG index increased. Second, the male and female participants in the middle and highest tertiles of the TyG index were 1.775 and 3.369 or 1.993 and 3.157 times more likely to have sarcopenic obesity, respectively. Third, we identified the most appropriate cut-off values of the TyG index for the identification of sarcopenic obesity in males and females as ≥8.72 and 8.67, respectively. However, due to the low AUCs (sensitivity and specificity), further studies including other cohort groups are required to determine the predictability of the TyG index for sarcopenic obesity. 

## Figures and Tables

**Table 1:** Characteristics of the study participants and differences between males and females.

	Overall (*n* = 3821)	Males (*n* = 1636)	Females (*n* = 2185)	*p* Value
TyG index	8.70 ± 0.53	8.71 ± 0.53	8.70 ± 0.52	0.834
Sarcopenia index	27.83 ± 4.28	27.88 ± 4.32	27.79 ± 4.25	0.520
Body mass index, kg/m^2^	23.8 ± 3.2	23.8 ± 3.1	23.8 ± 3.2	0.784
Age, years	69.2 ± 6.0	69.1 ± 5.9	69.4 ± 6.1	0.155
Height, cm	158.0 ± 8.9	158.3 ± 8.8	157.7 ± 9.0	<0.05
Body mass, kg †	59.6 ± 10.0	59.8 ± 9.7	59.5 ± 10.3	0.242
ASM, kg	16.61 ± 3.92	16.70 ± 3.88	16.55 ± 3.95	0.244
Glucose, mg/dL †	101.8 ± 21.5	102.2 ± 22.2	101.5 ± 21.0	0.941
Triglyceride, mg/dL	135.4 ± 68.9	135.4 ± 69.0	135.4 ± 68.9	0.999

Values are mean ± SD. † The Mann–Whitney U test was used to identify differences between groups. Sarcopenia index = (ASM/body mass) ×100. Abbreviations: TyG index = triglyceride–glucose index; ASM = appendicular skeletal muscle mass; SD, standard deviation.

**Table 2:** Sex-specific differences and trends of participants by TyG index tertile.

	A The Lowest	B The Middle	C The Highest	Post-Hoc	SS	*P* forTrend ‡
**Males**						
*n*	542	551	543			
TyG index †	8.12 ± 0.23 (8.10, 8.14)	8.69 ± 0.14 (8.67, 8.70)	9.31 ± 0.25 (9.29, 9.33)	A < B < C	42.89	<0.001
Sarcopenia index †	28.86 ± 4.38 (28.49, 29.23)	27.63 ± 4.44 (27.26, 28.01)	27.15 ± 3.96 (26.82, 27.49)	A > B, C	−6.30	<0.001
BMI, kg/m^2^ †	23.0 ± 3.3 (22.7, 23.3)	23.9 ± 3.1 (23.6, 24.1)	24.6 ± 2.9 (24.3, 24.8)	A < B < C	8.43	<0.001
Age, years	68.9 ± 6.0 (68.4, 69.4)	69.4 ± 5.9 (68.9, 69.9)	68.9 ± 5.9 (68.4, 69.4)	NS	0.11	0.914
Height, cm †	158.9 ± 9.0 (158.1, 159.6)	158.1 ± 8.3 (157.4, 158.8)	158.1 ± 9.1 (157.3, 158.8)	NS	−1.56	0.119
Body mass, kg	58.2 ± 10.1 (57.3, 59.0)	59.8 ± 9.3 (59.0, 60.6)	61.5 ± 9.4 (60.7, 62.3)	A < B < C	5.59	<0.001
ASM, kg	16.78 ± 3.84 (16.46, 17.10)	16.55 ± 3.87 (16.22, 16.87)	16.77 ± 3.94 (16.44, 17.10)	NS	−0.18	0.859
FPG, mg/dL †	94.4 ± 13.1 (93.3, 95.5)	99.7 ± 16.8 (98.3, 101.1)	112.5 ± 29.4 (110.0, 114.9)	A < B < C	14.33	<0.001
TG, mg/dL †	74.0 ± 17.4 (72.5, 75.4)	122.3 ± 23.4 (120.4, 124.3	210.1 ± 63.0 (204.8, 215.4)	A < B < C	39.68	<0.001
**Females**						
*n*	727	726	732			
TyG index †	8.12 ± 0.24 (8.11, 8.14)	8.69 ± 0.13 (8.68, 8.70)	9.29 ± 0.26 (9.27, 9.31)	A < B < C	49.57	<0.001
Sarcopenia index †	28.70 ± 4.47 (28.37, 29.03)	27.51 ± 4.17 (27.21, 27.82)	27.16 ± 3.94 (26.87, 27.45)	A > B, C	−6.46	<0.001
BMI, kg/m^2^	22.7 ± 3.2 (22.5, 23.0)	24.0 ± 3.1 (23.7, 24.2)	24.8 ± 3.0 (24.6, 25.1)	A < B < C	12.73	<0.001
Age, years	69.6 ± 6.0 (69.2, 70.0)	69.1 ± 6.0 (68.6, 69.5)	69.4 ± 6.2 (68.9, 69.8)	NS	−0.77	0.441
Height, cm	157.6 ± 9.1 (157.0, 158.3)	157.4 ± 8.9 (156.7, 158.0)	158.2 ± 8.8 (157.5, 158.8)	NS	0.92	0.356
Body mass, kg	56.6 ± 10.2 (55.9, 57.4)	59.5 ± 10.0 (58.8, 60.3)	62.3 ± 10.0 (61.6, 63.0)	A < B < C	10.99	<0.001
ASM, kg †	16.23 ± 3.82 (15.95, 16.51)	16.41 ± 3.92 (16.12, 16.70)	17.00 ± 4.08 (16.71, 17.30)	A, B < C	3.56	<0.001
FPG, mg/dL †	94.6 ± 12.3 (93.7, 95.5)	99.5 ± 16.2 (98.4, 100.7)	110.4 ± 27.8 (108.4, 112.4)	A < B < C	15.74	<0.001
TG, mg/dL †	73.9 ± 16.8 (72.7, 75.1)	122.1 ± 21.0 (120.5, 123.6)	209.8 ± 63.0 (205.2, 214.3)	A < B < C	46.55	<0.001

Values are the mean ± SD (95% CI). † The Mann–Whitney U test was used to identify differences between groups. ‡ The Jonckheere–Terpstra test was used to identify trends across the three groups. Sarcopenia index = (ASM/body mass) × 100. Abbreviations: SS = standardized statistic; TyG index = triglyceride–glucose index; BMI = body mass index; NS = not significant; ASM = appendicular skeletal muscle mass; FPG = fasting plasma glucose; TG = triglycerides; CI = confidence interval.

## Data Availability

The datasets analyzed in the current study are available from the corresponding author on reasonable request.

## Supplementary Materials

The following supporting information can be downloaded at: https://www.mdpi.com/article/10.3390/nu15030555/s1. Table S1: Characteristics of the young reference group, Table S2: Characteristics and comparisons of study participants, Table S3: The sex-specific differences in and trends of participants by TyG index tertile.
