# Supplementary material for: Triglyceride–Glucose Index as a Potential Indicator of Sarcopenic Obesity in Older People
Source: Nutrients. 2023 Jan 20;15(3):555. doi: 10.3390/nu15030555 (PMC9920571; doi:10.3390/nu15030555)
Supplement: Supplementary file 1 [file nutrients-15-00555-s001.zip › nutrients-2124545-supplementary.pdf]

Table S1. Characteristics of young reference group.

	Overall (n = 4918)	Males (n = 1910)	Females (n = 3008)	*P* Value
Sarcopenia index†	28.89 ± 4.27	33.10 ± 2.92	26.22 ± 2.47	< 0.001
Age, year	31.4 ± 5.5	31.3 ± 5.5	31.5 ± 5.5	0.261
Height, cm†	165.4 ± 8.5	173.4 ± 5.8	160.4 ± 5.5	< 0.001
Body weight, kg†	62.8 ± 12.9	72.4 ± 11.5	56.7 ± 9.6	< 0.001
Body mass index, kg/m^2	22.8 ± 3.6	24.1 ± 3.4	22.0 ± 3.5	< 0.001
Waist circumstance, cm	77.25 ± 10.28	82.75 ± 9.38	73.75 ± 9.25	< 0.001
ASM, kg	18.29 ± 5.18	23.81 ± 3.32	14.79 ± 2.29	< 0.001

Values are means ± SD. †Mann-Whitney U test was applied to assess the difference between groups. Sarcopenia index = (ASM/body mass) × 100; ASM = Appendicular skeletal muscle mass.

Table S2. Characteristics and comparisons of study participants.

	Overall (n = 3821)	Males (n = 1636)	Females (n = 2185)	*P* Value
ALM, kg	17.56 ± 4.17	17.66 ± 4.13	17.49 ± 4.20	0.227
ABM, kg	0.95 ± 0.29	0.96 ± 0.29	0.94 ± 0.29	0.113
WBFM, kg	17.09 ± 5.77	17.12 ± 5.71	17.06 ± 5.82	0.726
WBLM, kg	42.04 ± 8.28	42.18 ± 8.16	41.92 ± 8.36	0.340
%FM, %	28.78 ± 8.08	28.77 ± 8.15	28.78 ± 8.04	0.974
SBP, mmHg	130.9 ± 17.6	130.5 ± 17.3	131.2 ± 17.9	0.267
DBP, mmHg	77.9 ± 9.2	77.9 ± 9.9	78.0 ± 9.9	0.789
TC, mg/dL	191.5 ± 36.5	191.7 ± 36.4	191.4 ± 36.6	0.795
HDL, mg/dL	46.2 ± 10.9	46.2 ± 10.7	46.2 ± 11.1	0.920
AST, U/L[†]	24.0 ± 11.4	23.9 ± 12.0	24.1 ± 10.9	0.711
ALT, U/L[†]	20.6 ± 13.1	20.4 ± 13.4	20.8 ± 12.9	0.455
Creatinine, mg/dL	0.841 ± 0.251	0.842 ± 0.276	0.840 ± 0.231	0.826
Nutrition				
TEI, kcal/d	1718.8 ± 667.5	1727.6 ± 666.6	1712.2 ± 668.2	0.480
Carbohydrate, g	310.4 ± 112.8	310.1 ± 108.7	310.5 ± 115.7	0.913
Protein, g[†]	57.3 ± 30.5	58.1 ± 31.5	56.7 ± 29.8	0.293
Fat, g	24.6 ± 22.7	24.8 ± 20.9	24.5 ± 23.9	0.709
Medication (%)				
Hypertension	1691 (44.3)	657 (40.2)	1034 (47.3)	< 0.001
Dyslipidemia	358 (9.4)	107 (6.5)	251 (11.5)	< 0.001
Medical history (%)				
Stroke	201 (5.3)	101 (6.2)	100 (4.6)	< 0.05
CVD	239 (6.3)	124 (7.6)	115 (5.3)	< 0.01
Cancer	395 (10.3)	134 (8.2)	261 (12.0)	< 0.001
Liver disease	74 (1.9)	42 (2.6)	32 (1.5)	< 0.05
Arthritis	1414 (37.0)	297 (18.2)	1117 (51.1)	< 0.001
Phthisis	360 (9.4)	212 (13.0)	148 (6.8)	< 0.001
Asthma	275 (7.2)	111 (6.8)	164 (7.5)	0.411
Atopic dermatitis	73 (1.9)	27 (1.7)	46 (2.1)	0.341
MVPA (%)				0.723
Low (< 150 min/week)	2979 (78.0)	1271 (77.7)	1708 (78.2)	
High (≥ 150 min/week)	842 (22.0)	365 (22.3)	477 (21.8)	
Household income (%)				< 0.001
Low	1778 (46.5)	675 (41.3)	1103 (50.5)	
Lower middle	977 (25.6)	448 (27.4)	529 (24.2)	
Upper middle	610 (16.0)	290 (17.7)	320 (14.6)	
High	456 (11.9)	223 (13.6)	233 (10.7)	
Education level (%)				< 0.001
Primary school	2512 (65.7)	742 (45.4)	1770 (81.0)	
Middle school	529 (13.8)	331 (20.2)	198 (9.1)	
High school	536 (14.0)	367 (22.4)	169 (7.7)	
College	244 (6.4)	196 (12.0)	48 (2.2)	
Alcohol consumption (%)				< 0.001
No drink for last a year	1860 (48.7)	484 (29.6)	1376 (63.0)	
≤ once a week	1252 (32.8)	541 (33.1)	711 (32.5)	
2-3 times/week	322 (8.4)	263 (16.1)	59 (2.7)	

≥ 4 times/week	387 (10.1)	348 (21.3)	39 (1.8)	
Current smoking (%)				< 0.05
Never	1095 (28.7)	508 (31.1)	587 (26.9)	
Former smoking	490 (12.8)	202 (12.3)	288 (13.2)	
Current smoking	2236 (58.5)	926 (56.6)	1310 (60.0)	

Values are means ± SD. †Mann-Whitney U test was applied to assess the difference between groups. ALM = appendicular lean mass; ABM = appendicular bone mass; WBFM = whole body fat mass; WBLM = whole body lean mass; %FM = percentage of fat mass; SBP = systolic blood pressure; DBP = diastolic blood pressure; TC = total cholesterol; HDL = high density lipoprotein; AST = Aspartate transaminase; ALT = alanine transaminase; TEI = total energy intake; CVD = cardiovascular disease; MVPA = moderate to vigorous physical activity.

Table S3. The sex-specific differences and trends of participants by TyG Index tertile.

	A<br>**The lowest**	B<br>**The middle**	C<br>**The highest**	**Post-hoc**	**SS[‡]**	***P* for trend[‡]**
**Men**						
n	542	551	543			
ALM, kg	17.74 ± 4.10<br>(17.40, 18.09)	17.49 ± 4.10<br>(17.15, 17.83)	17.74 ± 4.19<br>(17.39, 18.10)	NS	-0.11	0.909
ABM, kg	0.96 ± 0.30<br>(0.94, 0.99)	0.94 ± 0.28<br>(0.92, 0.97)	0.97 ± 0.30<br>(0.95, 1.00)	NS	0.58	0.565
WBFM, kg[†]	15.60 ± 5.97<br>(15.09, 16.10)	17.49 ± 5.71<br>(17.01, 17.96)	18.28 ± 5.08<br>(17.85, 18.71)	A < B < C	7.92	< 0.001
WBLM, kg	42.10 ± 8.20<br>(41.40, 42.79)	41.81 ± 8.01<br>(42.14, 42.48)	42.65 ±8.28<br>(41.95, 43.34)	NS	1.01	0.314
%FM, %[†]	26.80 ± 8.47<br>(26.08, 27.51)	29.42 ± 8.22<br>(28.73, 30.11)	30.09 ± 7.35<br>(29.47, 30.71)	A < B, C	6.36	< 0.001
SBP, mm Hg	128.6 ± 17.6<br>(127.1, 130.0)	130.2 ± 17.2<br>(128.7, 131.6)	132.9 ± 16.9<br>(131.5, 134.3)	A, B < C	4.47	< 0.001
DBP, mm Hg	76.9 ± 9.8<br>(76.1, 77.7)	78.3 ± 10.1<br>(77.5, 79.1)	78.5 ± 9.9<br>(77.6, 79.3)	A < C	2.73	< 0.01
TC, mg/dL[†]	182.6 ± 33.0<br>(179.8, 185.4	193.2 ± 34.7<br>(190.3 196.1)	199.4 ± 39.3<br>(196.1, 202.7)	A < B < C	7.37	< 0.001
HDL, mg/dL[†]	50.5 ± 10.7<br>(49.6, 51.4)	46.3 ± 10.0<br>(45.5, 47.2)	41.8 ± 9.7<br>(41.0, 42.6)	A > B > C	-14.01	< 0.001
AST, U/L[†]	23.9 ± 12.8<br>(22.8, 25.0)	22.9 ± 8.2<br>(22.3, 23.6)	25.0 ± 14.2<br>(23.8, 26.2)	B < C	0.74	0.461
ALT, U/L[†]	18.5 ± 9.8<br>(17.7, 19.4)	19.4 ± 10.0<br>(18.6, 20.3)	23.3 ± 18.2<br>(21.8, 24.8)	A, B < C	6.45	< 0.001
Crea, mg/dL	0.841 ± 0.354<br>(0.811, 0.871)	0.835 ± 0.255<br>(0.813, 0.856)	0.851 ± 0.197<br>(0.835, 0.868)	NS	2.12	< 0.05
TEI, kcal/d	1723.4 ± 629.0<br>(1670.3, 1776.5)	1726.1 ± 670.9<br>(1670.0, 1782.2	1733.5 ± 699.1<br>(1674.5 1792.4)	NS	-0.40	0.692
Cabo, g	311.9 ± 106.1<br>(303.0, 320.9)	310.0 ± 109.6<br>(300.9, 319.2)	308.4 ± 110.5<br>(299.1, 317.7)	NS	-0.52	0.602
Protein, g	57.8 ± 29.9<br>(55.2, 60.3)	59.5 ± 34.5<br>(56.6, 62.4)	57.1 ± 29.9<br>(54.6, 59.7)	NS	-0.44	0.658
Fat, g	25.2 ± 21.8<br>(23.3, 27.0)	24.6 ± 20.9<br>(22.8, 26.3)	24.5 ± 19.9<br>(22.8, 26.2)	NS	-0.64	0.523
**Women**						
n	727	726	732			
ALM, kg	17.16 ± 4.06<br>(16.86, 17.45)	17.35 ± 4.18<br>(17.04, 17.65)	17.97 ± 4.33<br>(17.66, 18.28)	A, B < C	3.50	< 0.001
ABM, kg	0.93 ± 0.28<br>(0.91, 0.95)	0.94 ± 0.29<br>(0.92, 0.96)	0.97 ± 0.29<br>(0.95, 0.99)	A < C	2.41	< 0.05
WBFM, kg[†]	15.26 ± 6.04<br>(14.82, 15.70)	17.37 ± 5.68<br>(16.96, 17.79)	18.53 ± 5.23<br>(18.15, 18.91)	A < B < C	10.90	< 0.001
WBLM, kg	40.91 ± 8.06<br>(40.32, 41.50)	41.64 ± 8.28<br>(41.03, 42.24)	43.22 ± 8.57<br>(42.60, 43.84)	A, B < C	5.18	< 0.001
%FM, %[†]	26.87 ± 8.56<br>(26.25, 27.49)	29.36 ± 7.95<br>(28.78, 29.94)	30.11 ± 7.19<br>(29.59, 30.63)	A < B, C	7.14	< 0.001
SBP, mm Hg	128.5 ± 18.8<br>(127.1, 129.8)	131.5 ± 17.8<br>(130.2, 132.7)	133.6 ± 16.6<br>(132.4, 134.8)	A < B, C	5.74	< 0.001
DBP, mm Hg	76.4 ± 10.2	77.7 ± 9.6	79.8 ± 9.6	A < B < C	6.48	< 0.001

	(75.7, 77.2)	(77.0, 78.4)	(79.1, 80.5)			
TC, mg/dL†	183.6 ± 34.6<br>(181.1, 186.1)	189.5 ± 34.9<br>(186.9, 192.0)	200.9 ± 38.2<br>(198.2, 203.7)	A < B < C	8.51	< 0.001
HDL, mg/dL†	51.6 ± 11.9<br>(50.8, 52.5)	45.6 ± 9.7<br>(44.9, 46.3)	41.5 ± 9.2<br>(40.9, 42.2)	A > B > C	-18.16	< 0.001
AST, U/L†	23.9 ± 10.0<br>(23.2, 24.6)	23.2 ± 9.4<br>(22.6, 23.9)	25.1 ± 12.9<br>(24.2, 26.0)	A > B, B < C	0.95	0.345
ALT, U/L†	18.9 ± 11.1<br>(18.1, 19.7)	19.9 ± 11.6<br>(19.0, 20.7)	23.4 ± 15.2<br>(22.3, 24.5)	A < B < C	8.62	< 0.001
Crea, mg/dL	0.813 ± 0.206<br>(0.798, 0.828)	0.841 ± 0.255<br>(0.823, 0.860)	0.866 ± 0.225<br>(0.850, 0.883)	A, B < C	4.98	< 0.001
TEI, kcal/d	1731.9 ± 649.7<br>(1684.5, 1779.2)	1639.7 ± 655.6<br>(1645.9, 1741.5)	1711.2 ± 698.3<br>(1660.5, 1761.8)	NS	-0.95	0.343
Cabo, g	313.1 ± 120.9<br>(304.3, 321.9)	310.2 ± 115.3<br>(301.8, 318.6)	308.3 ± 110.8<br>(300.3, 316.4)	NS	-0.60	0.547
Protein, g	58.5 ± 31.2<br>(56.2, 60.8)	55.2 ± 27.8<br>(53.2, 57.2)	56.5 ± 30.2<br>(54.3, 58.6)	NS	-1.23	0.218
Fat, g	25.3 ± 22.8<br>(23.6, 26.9)	23.5 ± 19.5<br>(22.1, 24.9)	24.7 ± 28.5<br>(22.7, 26.8)	NS	-1.48	0.139

Values are means ± SD (95% CI). †Mann-Whitney U test was applied to assess the difference between groups. ‡Jonckheere-Terpstra test was used to assess the trend among three groups. SS = standardized statistic; ALM = Appendicular lean mass; ABM = Appendicular bone mass; WBFM = Whole body fat mass; WBLM = Whole body lean mass; %FM = percentage of fat mass; SBP = systolic blood pressure; DBP = diastolic blood pressure; TC = Total cholesterol; HDL = high density lipoprotein cholesterol; AST = Aspartate transaminase; ALT = alanine transaminase; Crea = Creatinine; TEI = Total energy intake; Carbo = Carbohydrate.

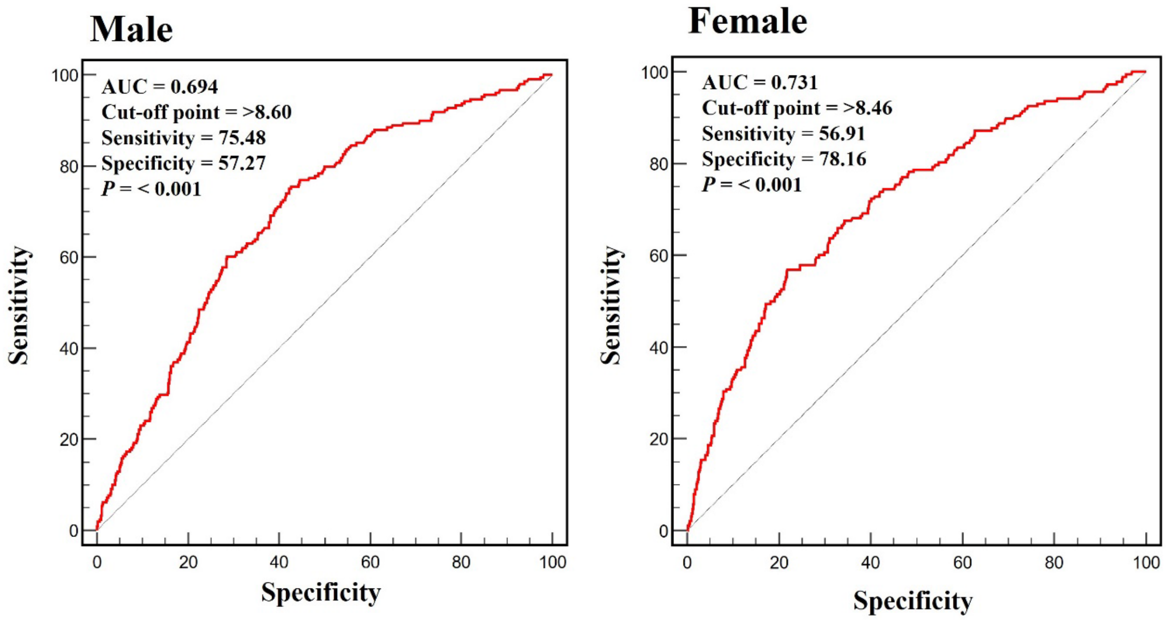

Figure S1. Sex-specific ROC curves pertaining to the TyG index for the Sarcopenic obesity group among the young reference group. Dotted blue line: reference; solid red line: AUC, indicative of the accuracy of the use of the TyG index for the identification of sarcopenic obesity; cut-off value: the value of the TyG index that predicts sarcopenic obesity; sensitivity: the probability of individuals who actually have sarcopenic obesity being identified as having sarcopenic obesity; specificity: the probability of individuals who do not have sarcopenic obesity being identified as not having sarcopenic obesity. Abbreviations: AUC, the area under the curve; ROC, receiver operating characteristic.
